# Coordinated control of Notch/Delta signalling and cell cycle progression drives lateral inhibition-mediated tissue patterning

**DOI:** 10.1242/dev.134213

**Published:** 2016-07-01

**Authors:** Ginger L. Hunter, Zena Hadjivasiliou, Hope Bonin, Li He, Norbert Perrimon, Guillaume Charras, Buzz Baum

**Affiliations:** 1 MRC-Laboratory for Molecular and Cell Biology, University College London, London WC1E 6BT, UK; 2 Institute of Physics of Living Systems, University College London, London WC1E 6BT, UK; 3 Centre for Mathematics, Physics, and Engineering in the Life Sciences and Experimental Biology, University College London, London WC1E 6BT, UK; 4 Department of Genetics, Evolution and Environment, University College London, London WC1E 6BT, UK; 5 Howard Hughes Medical Institute, Department of Genetics, Harvard Medical School, Boston, MA 02115, USA; 6 London Centre for Nanotechnology, University College London, London WC1E 6BT, UK; 7 Department of Cell and Developmental Biology, University College London, London WC1E 6BT, UK

**Keywords:** Notch signalling, Cell cycle, Lateral inhibition, Patterning, G2 phase

## Abstract

Coordinating cell differentiation with cell growth and division is crucial for the successful development, homeostasis and regeneration of multicellular tissues. Here, we use bristle patterning in the fly notum as a model system to explore the regulatory and functional coupling of cell cycle progression and cell fate decision-making. The pattern of bristles and intervening epithelial cells (ECs) becomes established through Notch-mediated lateral inhibition during G2 phase of the cell cycle, as neighbouring cells physically interact with each other via lateral contacts and/or basal protrusions. Since Notch signalling controls cell division timing downstream of Cdc25, ECs in lateral contact with a Delta-expressing cell experience higher levels of Notch signalling and divide first, followed by more distant neighbours, and lastly Delta-expressing cells. Conversely, mitotic entry and cell division makes ECs refractory to lateral inhibition signalling, fixing their fate. Using a combination of experiments and computational modelling, we show that this reciprocal relationship between Notch signalling and cell cycle progression acts like a developmental clock, providing a delimited window of time during which cells decide their fate, ensuring efficient and orderly bristle patterning.

## Abstract (precis)

**Summary:** In the fly notum, a reciprocal relationship between Notch signalling and cell cycle progression acts like a clock, providing a delimited window of time during which cells decide their fate.

## INTRODUCTION

**Fig. 1.**
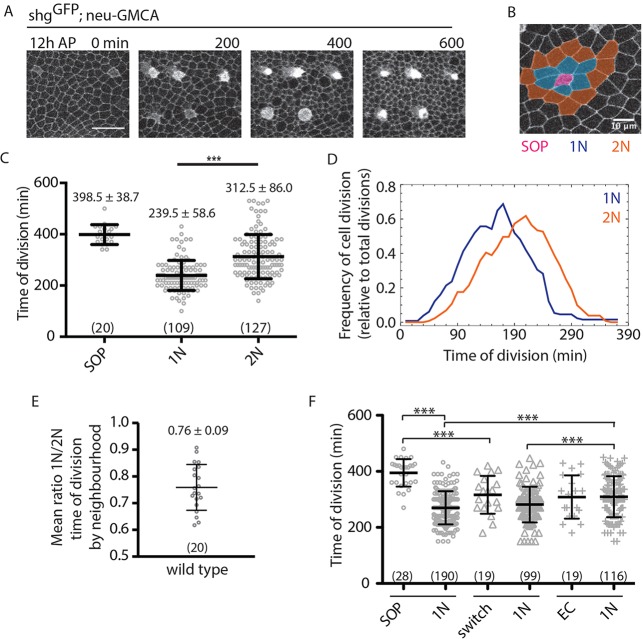
**Spatiotemporal patterning of notum cell divisions.** (A) Pupal notum expressing Shotgun^GFP^ (cell boundaries), and nGMCA (SOPs) over time. Posterior to the left, anterior to the right. Scale bar: 25 µm. (B) SOP ‘neighbourhood’: SOP (pink) with primary (1N; blue) and secondary (2N; orange) neighbours. Scale bar: 10 µm. (C) Time of cell division for the genotype shown in A; *N*=2 pupae. (D) Line graph of the data shown in C. (E) Mean ratio of local SOP neighbourhood division timing, genotype as in A. *N*=2 nota; *n*=20 SOPs, 109 1Ns, 127 2Ns. (F) Division timing of SOPs, ‘switch’ cells (neu-GMCA-expressing cells that switch to EC fate) and ECs and their respective 1Ns in shotgun^GFP^; neu-GMCA pupae (*N*=3). ***P<0.001 (unpaired two-tailed *t*-test for pairs indicated). Mean±s.d. shown. (*n*)=number of cells.

In the *Drosophila* notum, Notch-mediated lateral inhibition drives the emergence of a patterned array of microchaete, or small mechanosensory bristles, ∼8-18 h after pupariation (AP) at 25°C (Fig. 1A; Movie 1) (Simpson et al., 1999; Furman and Bukharina, 2008; Cohen et al., 2010). Cells with low levels of activated Notch signalling adopt a sensory organ precursor cell (SOP) fate, and divide to give rise to the microchaete lineage (Simpson, 1990). Moreover, SOPs express high levels of neural precursor genes and Delta ligand (Muskavitch, 1994; Parks et al., 1997), which activates Notch signalling in surrounding cells to prevent them from adopting a neural fate (Muskavitch, 1994). In this way, Notch/Delta signalling breaks symmetry to pattern the tissue (Parks et al., 1997). Notch signalling in this tissue is not limited to lateral cell contacts: a network of dynamic, actin-based protrusions at the basal side of the epithelium aids signal propagation over longer distances (de Joussineau et al., 2003; Cohen et al., 2010). This type of protrusion-mediated signalling (Hamada et al., 2014; Kornberg and Roy, 2014; Khait et al., 2016), it has been argued (Cohen et al., 2010, 2011), helps ensure the gradual emergence and refinement of a pattern of well-spaced SOPs.

Work across eukaryotic systems suggests that the decision to exit the cell cycle and divide often occurs in G1 (Vidwans and Su, 2001; Lee and Orr-Weaver, 2003). Nevertheless, some cell fate decisions, including the development of macrochaete (Usui and Kimura, 1992; Kimura et al., 1997; Nègre et al., 2003), appear to be made during passage through G2. In this paper, we show how feedback between cell fate-determining signals and progression through mitosis coordinates timely epithelial patterning in the fly notum.

## RESULTS AND DISCUSSION

During notum development, all ECs divide once (Bosveld et al., 2012) (Movie 1), before undergoing terminal differentiation. At the same time, an initially disordered array of cells expressing proneural genes is refined to generate an ordered pattern of bristles in adults (Cohen et al., 2010; Protonotarios et al., 2014) (Fig. 1A). By simultaneously following cell division and patterning in this tissue, we find that local patterns of division timing correlate with proximity to SOPs (Fig. 1B-D). ECs sharing long cell-cell interfaces with SOPs, hereafter termed primary neighbours (1N), divide first. These are followed by next-nearest ECs, or secondary neighbours  (2N), which contact SOPs via dynamic basal protrusions alone (Cohen et al., 2010). SOPs divide last (Fig. 1C). The local spatiotemporal pattern of divisions is robust, as indicated by a ratio of division times for neighbours surrounding each SOP of <1 (Fig. 1E), even though the timing of bristle-row patterning is developmentally staggered (Usui and Kimura, 1993; Parks et al., 1997). Moreover, ECs that transiently express proneural markers (Cohen et al., 2010) (Fig. S1A-C), including Delta (Kunisch et al., 1994), before assuming an EC fate accelerate G2 exit in their EC neighbours (Fig. 1F).

### The local pattern of EC division is Notch dependent

**Fig. 2.**
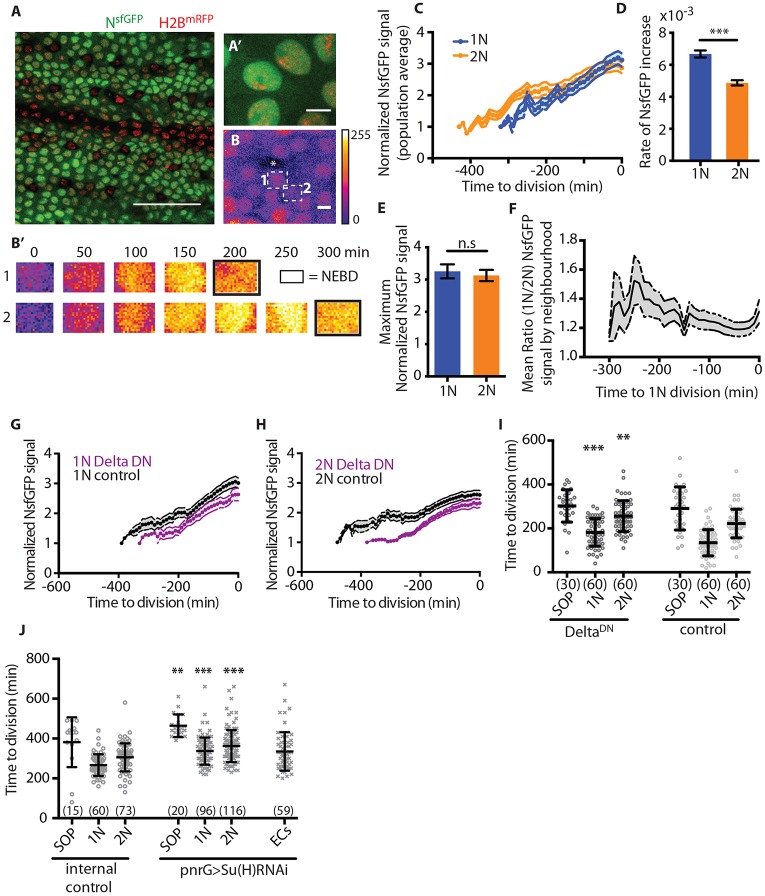
**Cell division timing depends on Notch signalling.** (A) N^sfGFP^ expression pattern at 12 h AP. H2B^mRFP^ labels nuclei. Scale bar: 50 µm. (A′) Higher magnification image of A. Scale bar: 5 µm. (B) False-coloured panel of N^sfGFP^ -expressing ECs. Asterisk indicates SOP. Primary (1) and secondary (2) neighbours are indicated by dashed boxes. Scale bar: 5 µm. (B′) Time series of nuclear ROIs for cells 1 and 2 until nuclear envelope breakdown (NEBD; indicated by black boxes), leading to transient depletion of signal. (C) N^sfGFP^ dynamics in ECs (*n*=29 each, *N*=3). (D) Rate of N^sfGFP^ increase for the data shown in C. (E) Maximum normalized N^sfGFP^ signal for the data shown in C. (F) Mean ratio of local SOP neighbourhood N^sfGFP^ signal (*n*=27 SOP, 133 each EC type; *N*=3). (G-I) neur-GAL4 expression of Delta^DN^ reduces Notch signalling in wild-type 1N (G) or 2N (H) cells (*n*=16, *N*=2) versus control (UAS-lifeActRuby, *n*=30, *N*=3) and delays cell division timing in Shotgun^GFP^; neu-GAL4, UAS-GMCA>UAS-Delta^DN^ pupae (I) (*N*=3). (J) Cell division timing in Shotgun^GFP^; pnrGAL4>UAS-Su(H) RNAi pupae relative to control (*N*=2). ECs, epithelial cells in regions lacking differentiating SOPs. Mean±s.e.m. for C,F,G,H; mean±s.d. for D,E,I,J. n.s., not significant; **P<0.01; ***P<0.0001 by unpaired, two-tailed, *t*-test as indicated compared with control of the same type (i.e. RNAi-1N to control-1N). (*n*)=number of cells.

If lateral inhibition cues division timing, as suggested by these observations, we can make the following predictions. First, for each SOP neighbourhood, there should be differences in the intensity of Notch signalling between primary and secondary neighbours. Second, perturbing Notch signalling should disrupt the pattern of cell divisions. To test this, we visualized signalling dynamics using *Notch-nls:sfGFP* (N^sfGFP^) (Fig. 2A,B). N^sfGFP^ is a nuclear localized, PEST-tagged (unstable), super-folder GFP expressed downstream of a minimal GBE-Su(H) promoter (L.H. and N.P., unpublished) (Li et al., 1998; Furriols and Bray, 2001) (Fig. S1A-C).

At 12 h AP, N^sfGFP^ is visible in EC rows in which bristle formation occurs (Fig. S1A) (Usui and Kimura, 1993). Notch signalling increases nearly linearly in ECs until division (Fig. 2C; Fig. S1D-G). The rate of response, which functions as a measure of signal strength, is higher in primary than secondary neighbours (Fig. 2C,D). The peak N^sfGFP^ signal is similar for both neighbours when measured across the tissue (Fig. 2E). However, the local ratio of N^sfGFP^ signal prior to division is >1 (Fig. 2F), suggesting that primary ECs receive a higher Delta signal from individual SOPs than do secondary ECs.

To test whether N^sfGFP^ signal and division timing in ECs depends on Delta expression in SOPs, we measured local N^sfGFP^ signal following laser ablation of SOPs (Fig. S1H). Under these conditions, N^sfGFP^ signal accumulation halts in primary and secondary ECs, but continues to increase in ECs proximal to both the wound and intact SOPs (Fig. S1I), as expected if the signal depends on a Delta input from the ablated SOP. Relative to controls, EC divisions are delayed following local SOP loss (Fig. S1J). Additionally, we found that dominant-negative Delta ligand (Delta^DN^) overexpression in SOPs decreases N^sfGFP^ signal in neighbouring ECs (Fig. 2G,H) (Herranz et al., 2006). Together with the ablation data, this shows that N^sfGFP^ signal in ECs is dependent upon Delta-expressing SOPs.

Next, we examined the effects of disrupting Notch signalling on cell division timing by overexpressing Delta^DN^ in SOPs (Fig. 2I) or using RNAi against Suppressor of Hairless [Su(H)], an essential component of Notch-targeted gene expression (Lehman et al., 1999; Furriols and Bray, 2001) across the tissue. Delta^DN^ expression did not disrupt the pattern of local division timings but was sufficient to delay division of neighbouring ECs, as expected if Delta signal promotes division. Su(H) depletion blocks divisions within the *pnr* domain in the majority of animals (*N*=4/6 pupae), and later leads to tissue failure. In the remaining animals (*N*=2/6 pupae), which may express levels of Su(H) activity sufficient for tissue survival, divisions are delayed and the local pattern of divisions is perturbed in regions where microchaete are formed (Fig. 2J). Therefore, local cell division timing is dependent on Notch-mediated lateral inhibition.

### The local timing of EC division is dependent on Cdc25 and Wee1

At the onset of bristle patterning, cells in the notum are arrested in G2 of the cell cycle. All cells express a nuclear FUCCI-GFP marker (Fig. S2A) but do not stain for 5-ethynyl-2′-deoxyuridine (EdU), a marker for ongoing DNA replication (Fig. S2B). In many systems, G2 exit is regulated by the phosphatase Cdc25, encoded by *Drosophila string* (*stg*) (Edgar and O'Farrell, 1989; Courtot et al., 1992), which catalyses removal of an inhibitory phosphate group (added by the kinases Wee1 and Myt1; Price et al., 2000; Jin et al., 2008) from a regulatory tyrosine on Cdk1. Wee1 and Myt1 function in opposition to Cdc25 in many systems (Vidwans and Su, 2001), sometimes redundantly (Jin et al., 2008).

**Fig. 3.**
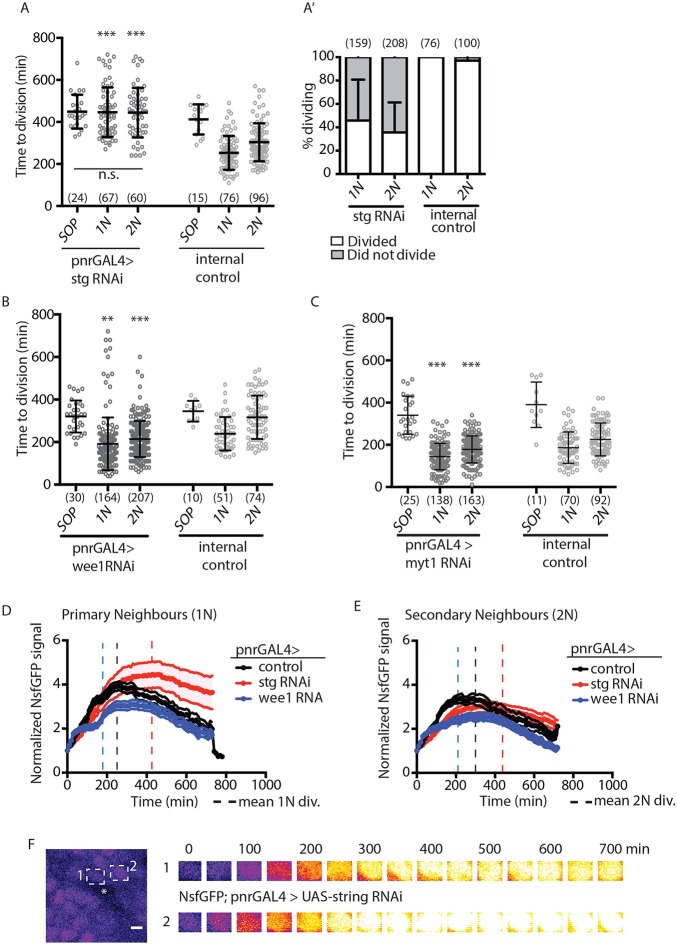
**Regulation of notum division timing.** (A) Cell division timing in Shotgun^GFP^; pnrGAL4>UAS-string RNAi pupae (*N*=3). n.s., not significant by one-way ANOVA. (A′) Percentage of dividing cells in the same genotype as A. (B,C) Cell division timing in Shotgun^GFP^; pnrGAL4>UAS-Wee1 RNAi (B) (*N*=3) or UAS-Myt1 RNAi (C) pupae (*N*=3). (D,E) N^sfGFP^ dynamics in primary (D) and secondary (E) neighbour ECs expressing UAS-stg RNAi (red; *n*=20, *N*=2), UAS-Wee1 RNAi (blue; *n*=20, *N*=2), or control (UAS-lifeActRuby, black; *n*=30, *N*=3) under pnr-GAL4. Vertical dashed lines indicate mean cell division timing for cell position and genotype. (F) Time series of nuclear ROIs for *string* RNAi-expressing cells 1 and 2 (indicated by dashed boxes), showing failure to downregulate signal. NEBD does not occur. Asterisk indicates SOP. **P≤0.01; ***P≤0.001 by unpaired, two-tailed, *t*-test to control of the same type. Mean±s.d. for A-C; mean±s.e.m. for D,E. (*n*)=number of cells.

To test whether Cdc25 and the kinases Wee1 and Myt1 regulate G2 exit in the notum, we expressed dsRNAs targeting these regulators under pnr-GAL4. *stg* RNAi expression delays EC division timing, prevents patterned divisions, and in some cases blocks division altogether (Fig. 3A,A′; Fig. S2C). Conversely, *wee1-* or *myt1* RNAi expression throughout the notum causes precocious EC entry into mitosis (Fig. 3B,C). Loss of *stg*, *wee1* or *myt1* expression does not affect the timing of the first division of SOPs (Fig. 3A-C) (which are subject to additional regulation; Ayeni et al., 2016). Together, these results support a model in which the opposing activities of Cdc25 and Wee1/Myt1 regulate EC division timing.

Conversely, the duration of G2 might influence Notch signalling. Because N^sfGFP^ decreases immediately after EC divisions, but prior to SOP division (Fig. 3D,E), we investigated whether division renders ECs refractory to Delta signal. To test this, we quantified N^sfGFP^ dynamics in cells in which the length of G2 was altered by *stg* RNAi or *wee1* RNAi. As expected if division curtails signalling, N^sfGFP^ expression was retained in cells with extended G2 (Fig. 3D-F), but was lost in those that divided prematurely (Fig. 3D,E). The timing of G2 exit appears to be crucial for a robust Notch response in ECs, which is terminated following division.

### Relative timing of SOP cell and EC division is crucial for bristle patterning

To examine the consequences of the observed coupling between Notch signalling and cell cycle progression on tissue patterning, we developed a mathematical model of lateral inhibition (see supplementary Materials and Methods for details) (Cohen et al., 2010; Sprinzak et al., 2010). The model follows the dynamics of transmembrane Notch receptor (*N*), Delta ligand (*D*) and intracellular Notch (*R*; i.e. activated Notch) in a 2D array of cells. We model basal protrusion-mediated signalling (relevant for 1N, 2N) and signalling mediated by apicolateral cell-cell contacts (relevant for 1N only). The level of apical and basal signalling is weighted by α_a_ and α_b_, respectively; we set α_a_>α_b_ following previous observations (Benhra et al., 2010; Cohen et al., 2010). To couple signalling and division, we allow cells to divide with a probability *p_d_* at any time step, as a function of *R*, so that:
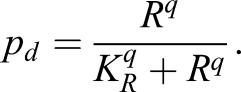


The value of *K_R_* determines the window of Notch response for which division becomes likely (Fig. S3A). To mimic events in the tissue, after division the developmental fate of a cell is locked and it no longer participates in lateral inhibition.

**Fig. 4.**
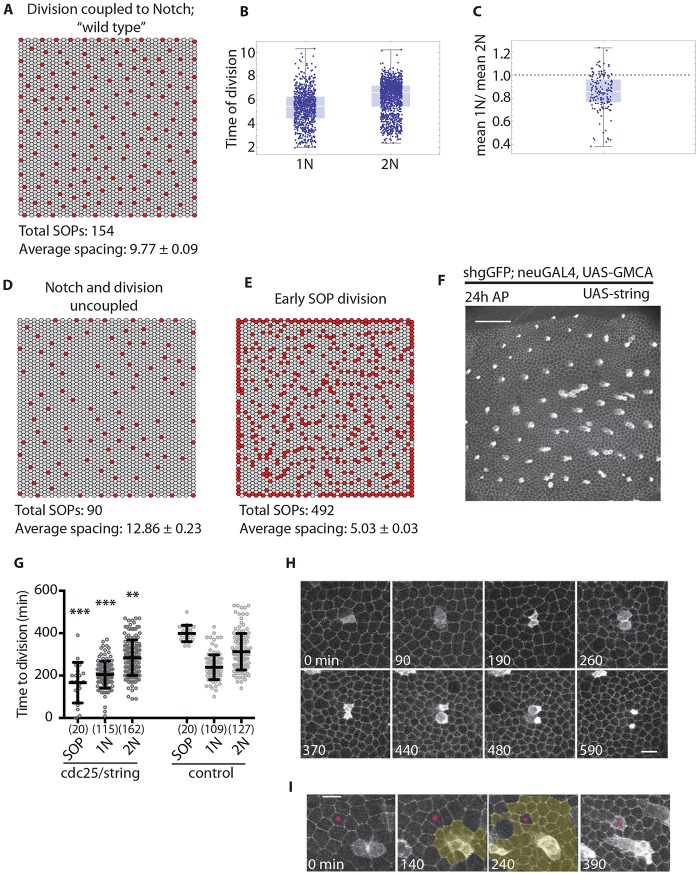
**Cell division timing is crucial for SOP patterning.** (A) Model output for ‘wild type’ simulation (*K_R_*=200, *q*=5). Average spacing is the mean±s.e.m. distance between each SOP (red) and its ten nearest SOPs. (B) Simulation results for cell division timing in 1N and 2N for the wild-type model described in A. (C) Ratio of mean time of division for 1N and 2N in the model. (D) Model output when Notch signalling and division timing are uncoupled, *p_d_*=0.005 (any non-Delta cell [*D*<1] divides with probability *p_d_*). (E) Model output when SOPs are forced to divide early (Delta cells [*D*>1] divide with probability *p_d_*=0.0001). Red, Delta expressing SOPs (*D*>1); grey, Notch-expressing ECs. (F) Final SOP pattern in tissues with precocious SOP division. Scale bar: 50 µm. (G) Cell division timing in Shotgun^GFP^; neu-GAL4, UAS-GMCA>UAS-string pupae (*N*=3, mean±s.d.). Control: Shotgun^GFP^; neu-GAL4, UAS-GMCA (*N*=3). (H,I) SOP ‘twins’ and secondary neighbour cell switching (asterisks in I), as a consequence of the precocious SOP division shown in F. Yellow, divided cells. Scale bars: 10 µm. **P≤0.01, ***P≤0.001, unpaired, two-tailed, *t*-test to control of same type. (*n*)=number of cells.

To model a wild-type tissue in which Notch signalling drives EC division, we set *q*=5 and *K_R_*=200 (Fig. 4A; Fig. S3B-D). Under these conditions, primary neighbours divide first, followed by secondary neighbours (Fig. 4B,C), consistent with spatiotemporal patterning of EC division *in vivo* (Fig. 1C-E); this delay persists even when *α*_a_=*α*_b_ (i.e. amount of apical or basal Delta is equivalent; Fig. S3E). The overall profile of Notch expression at division in neighbours generated by the model (Fig. S3D) is comparable to that seen *in vivo* (Fig. S1D-G). At the tissue level, the time taken to reach a stable pattern increases with *K_R_* (Fig. S3F), suggesting that for a given developmental time window, there is an optimal range of Notch response for determining cell fate.

Using this model, we tested the effect of uncoupling EC division timing from Notch signalling: any (non-Delta) cell may divide with a fixed probability *p_d_*, that is independent of Notch. This leads to sparse patterns with few Delta cells, particularly for large values of *p_d_* (Fig. 4D; Fig. S3G). We also tested the effect of primary and secondary neighbours dividing at the same time (Fig. S3H) by only allowing uniform protrusion-based signalling – where signal strength is independent of protrusion length. Under these conditions the pattern is again ordered but sparse. Together, this suggests that the delay in division in cells with low Notch expression is important for patterning. Because patterning is not uniform across the notum, this delay (Fig. 1C; Fig. 4B,C) preserves a pool of ECs that, because they lie far from SOPs and receive a weak Delta-input signal (Fig. 2D-F), have the potential to switch fate to help refine the bristle pattern as it emerges (Movie 1).

Next, we investigated the impact of changing the relative timing of SOP and EC divisions. When we couple Delta expression to a fixed value of *p_d_*, so that cells for which Delta expression exceeds a threshold (*D_th_*) can divide, clusters of Delta-expressing cells form that disrupt the pattern (Fig. 4E). This is because, under the model, a Delta cell that divides no longer inhibits its neighbours from acquiring an SOP fate. To test whether we observe similar behaviour *in vivo*, we overexpressed String in SOPs (Fig. 4F-I). This disrupts tissue patterning in two ways. First, we observe cells expressing low levels of neuralized reporter dividing early. Frequently, one daughter cell develops into an SOP, and the other is inhibited from doing so, switching to EC cell fate (47.5%, *n*=61; *N*=3) or delaminating (9.8%). In other cases, both daughter cells form SOPs (26.2%; Fig. 4H) and paired bristles (Fig. S3I). Second, we observed secondary neighbours of early dividing SOPs adopting an SOP fate (Fig. 4I), as in the model, probably following the loss of protrusion-mediated Delta signalling at division. We note that this phenotype is also observed on occasion in wild-type tissue, and is consistent with the observation that precocious SOP division terminates Delta signalling, leading to reduced levels of N^sfGFP^ signal in surrounding ECs (Fig. S3J,K). These data further support our hypothesis that cell division signals the termination of lateral inhibition between SOPs and ECs.

### Conclusions

The results of our experimental analysis show that Notch signalling drives EC division in the notum, coupling patterning to cell cycle progression. As shown in simulations, this aids timely and orderly patterning by taking cells ‘out of the game’, so that the fate of ECs is sealed before SOPs divide. The effects of re-wiring the system can be seen by the induction of premature SOP divisions, which in both experiment and model leads to the formation of excess SOPs as the result of secondary ECs changing their fate. The delay in the division of secondary and tertiary ECs, which receive a relatively weaker Delta input from local SOPs, provides a population of cells with an indeterminate fate that can be used to fill in any gaps in the pattern as it emerges. This is key to pattern refinement. Through an extended G2 phase, the system has a delimited window of time during which Notch and Delta can pattern the tissue through lateral inhibition, before signal-induced entry into mitosis fixes the pattern, driving the process to completion.

## MATERIALS AND METHODS

### Fly strains

‘Wild type’ refers to control animals. See supplementary Materials and Methods for a full list of fly strains used.

### Microscopy

White pre-pupae were picked and aged to 12 h AP at 18°C. Live pupae were dissected as previously described (Zitserman and Roegiers, 2011). Live pupae were imaged on a Leica SPE confocal, 40× oil immersion objective (1.15 NA) at room temperature. Fixed nota were imaged on a Leica SPE3 confocal, 63× oil immersion objective (1.3 NA). Datasets were captured using Leica LSM AF software.

### Laser ablation

Ablations were performed with 730-nm multiphoton excitation from a Chameleon-XR Ti–Sapphire laser on a Zeiss Axioskop2/LSM510 (AIM, Zeiss). Post-ablation images were acquired as described above.

### Immunofluorescence

Nota of staged pupae fixed as previously described (Zitserman and Roegiers, 2011) (see supplementary Materials and Methods for further details). Primary antibodies were anti-GFP (1:1000; Abcam) and anti-Dlg (1:500; Developmental Studies Hybridoma Bank). Secondary antibodies were Alexa Fluor 488-conjugated anti-chicken and Alexa Fluor 568-conjugated anti-mouse (both 1:1000; Thermo Fisher Scientific). EdU staining was performed using the Click-iT EdU Imaging Kit (Thermo Fisher Scientific).

### Quantification

N^sfGFP^ signal was quantified as follows: unprocessed imaging data was imported into Fiji (ImageJ, NIH). Mean pixel value for a nuclear region of interest (ROI) was taken for each time point. Normalized N^sfGFP^ is relative to N^sfGFP^ signal at t_0_. For neighbourhood measurements, nuclear ROIs were taken and averaged for four or five primary and four or five secondary ECs per SOP in bristle row 2. Internal control measurements were made in the same animals, but outside the pnr domain. For cell division timing panels, t=0 min at ∼12 h AP. Resulting data were analysed using Prism (Graphpad) and using statistical tests as outlined in figure legends.

### Mathematical model

See supplementary Materials and Methods for a full description of the mathematical model.
